# Therapeutic Plasma Exchange in Pediatric Patients: A Multi‐Institutional Landscape Analysis

**DOI:** 10.1002/jca.70198

**Published:** 2026-10-19

**Authors:** Caitlin Raymond

**Affiliations:** ^1^ Department of Pathology and Laboratory Medicine University of Wisconsin–Madison Madison Wisconsin USA

**Keywords:** all‐cause mortality, ASFA guidelines, electronic health record, pediatric apheresis, pediatrics, therapeutic plasma exchange, TriNetX

## Abstract

Pediatric evidence for therapeutic plasma exchange (TPE) is largely small, single‐center cohorts; there is no prior large‐scale age‐stratified outcomes assessment. Using the TriNetX US Collaborative Network, we identified 3744 patients ≤ 17 years old undergoing an index TPE procedure, stratified into four age groups (≤ 1, 2–5, 6–12, and 13–17 years). ICD‐10‐CM diagnosis codes recorded 30 days prior to the index procedure were mapped to American Society for Apheresis Categories I–III indications; three high‐prevalence nonspecific codes were tracked separately. All‐cause mortality at 30 days, 90 days, and 1 year was assessed. All‐cause mortality decreased with increasing age at every time window: 30‐day mortality ranged from 16.8% (≤ 1 year) to 4.3% (13–17 years, *p* < 0.0001). Infant mortality risk was concentrated disproportionately in the first 90 days postprocedure. Sex distribution shifted from a slight male predominance in infancy and early childhood (55%–56% male) to a female predominance in adolescence (55% female, *p* < 0.0001). Leading specific indications were autoimmune encephalitis (10%), sepsis with multiorgan failure (9%), renal transplant rejection (6%), acute disseminated encephalomyelitis (5%), Guillain–Barré syndrome (5%), and myasthenia gravis (4%); nonspecific diagnosis codes were each individually more prevalent than any single ASFA‐mapped indication. This is the first large‐scale, age‐stratified, multi‐institutional characterization of pediatric TPE. We identified an inverse relationship between age and all‐cause mortality, a sex‐distribution shift in adolescence, and an indication landscape dominated by nonspecific diagnosis codes. These findings offer age‐stratified benchmarks for clinical counseling and hypothesis generation.

## 1 Introduction

Therapeutic plasma exchange (TPE) is an extracorporeal procedure in which a patient's plasma is separated from cellular blood components and removed, typically along with pathogenic autoantibodies, immune complexes, or other high‐molecular‐weight solutes, and replaced with a substitution fluid such as albumin or donor plasma [1]. The American Society for Apheresis (ASFA) maintains an evidence‐based categorization of apheresis indications—Categories I through IV—ranking conditions by the strength of evidence supporting TPE as first‐line, adjunctive, or unproven/ineffective therapy [2].

While TPE is well established in adult apheresis medicine, the pediatric evidence base is comparatively limited. Existing pediatric TPE literature consists almost entirely of single‐center, or occasionally two‐ to three‐center, retrospective cohorts, the majority drawn from pediatric intensive care units (PICUs), with a smaller number focused specifically on pediatric neurology referral populations. Reported cohorts range from as few as 22 to roughly 320 patients [3, 4, 5, 6, 7, 8, 9, 10, 11], alongside a smaller set of reports focused specifically on TPE performed in tandem with continuous renal replacement therapy and/or extracorporeal membrane oxygenation [12, 13, 14]. A 2018 critical review and proposed treatment algorithm for TPE in pediatric neurology synthesized much of this literature [15], pooling complication data across several UK and international pediatric cohorts and reporting perprocedure complication rates ranging from roughly 7% to 55% depending on cohort definition and case mix—underscoring both the heterogeneity of outcome reporting in this field and the absence of a single cohort large enough to generate stable, age‐stratified estimates. This is in spite of the fact that children undergoing TPE span an unusually wide range of developmental stages, body sizes, and indications: from critically ill neonates and infants to adolescents with adult‐pattern autoimmune disease, all of which plausibly carry very different risk profiles.

The closest existing precedent for a multi‐institutional approach is Weiss et al.'s analysis of the Pediatric Health Information System (PHIS) [16], which used administrative discharge data from 42 US children's hospitals (2003–2010). Their cohort combined two ascertainment strategies: hospitalizations with a primary discharge diagnosis of an ASFA Category I or II condition, and separately, all hospitalizations with a procedure code indicating receipt of TPE regardless of the associated diagnosis. Using the procedure‐defined subset they found that only 9.0% and 2.2% of hospitalizations with a TPE procedure code carried a primary discharge diagnosis of a Category I or II condition, respectively. However, this study was limited by the absence of age strata and broad outcome data, and the results of this study are no longer current.

The present analysis extends this procedure‐first approach considerably: It draws on a more contemporary, broader, and substantially larger network; captures diagnosis codes recorded across a 30‐day window prior to the index procedure rather than relying on a single primary discharge diagnosis; and generates age‐stratified demographic and mortality outcomes that Weiss et al. did not examine. The objectives of this study were threefold: (1) to characterize the demographic composition of the pediatric TPE population across four clinically motivated age strata (≤ 1, 2–5, 6–12, and 13–17 years); (2) to characterize the clinical indications for pediatric TPE within each age stratum, mapped to ASFA categories, and to quantify the prevalence of nonspecific diagnosis coding that could not be mapped to a defined ASFA indication; and (3) to determine all‐cause mortality at 30 days, 90 days, and 1 year following the index TPE procedure, overall and by age stratum. Together, these aims are intended to establish descriptive, age‐stratified benchmarks for pediatric TPE practice and outcomes that can inform clinical expectations, guide future prospective study design, and support more granular, claims‐based indication‐assignment methodology in apheresis research.

## 2 Methods

### 2.1 Data Source

This study used the TriNetX US Collaborative Network [17], a federated research network providing de‐identified electronic health record data from up to 72 participating US healthcare organizations. Because data remain at the originating institution, results are returned as aggregate counts and derived statistics rather than patient‐level extracts. Following the TriNetX Analyze Outcomes default, only patients whose index TPE procedure occurred within the 20 years before the query date (approximately from July 2006 to July 2026) were analyzed. Queries were run July 18, 2026.

### 2.2 Cohort Identification and Age Stratification

The pediatric cohort included all patients ≤ 17 years old at their first (index) TPE procedure, identified by CPT 36514 or ICD‐10‐PCS 6A551Z3/6A550Z3, yielding a base cohort of 3744 patients. The cohort was stratified a priori into four age strata: ≤ 1, 2–5, 6–12, and 13–17 years. The sum of stratum *N*'s (3845) differs slightly from the all‐cohort *N* (3744, 2.7%), consistent with known real‐time count variation across sequential TriNetX queries [17]; because all indication and mortality percentages use stratum‐internal denominators, this does not affect the findings reported.

### 2.3 Indication Assignment and ASFA Category Mapping

ICD‐10‐CM diagnosis codes recorded within 30 days prior to the index procedure were extracted and mapped to ASFA Categories I–III (Category IV was not mapped in this analysis) according to the ASFA Ninth Special Issue, the current edition at the time of writing [2]. Indications across ASFA Categories I–III were chosen based on how robustly they could be captured in an ICD‐10 code. Because many temporally proximate codes did not map cleanly to a specific indication, three additional nonspecific codes—G93 (other disorders of brain), N17–N19 (acute/chronic kidney disease), and T86 (transplant complications)—were tracked separately given their high prevalence and plausible relevance to the decision to pursue TPE.

### 2.4 Outcome Assessment: All‐Cause Mortality

All‐cause mortality (TriNetX “Deceased” flag or ICD‐10‐CM R99) was assessed at 30 days, 90 days, and 1‐year postindex using TriNetX's Analyze Outcomes module, which reports both risk and Kaplan–Meier survival probability. This analysis was performed for the overall cohort and each age stratum.

### 2.5 Statistical Analysis

This is a descriptive analysis. TriNetX‐generated counts, percentages, means ± SD, and medians (IQR) are reported; comparisons across strata are supported with Cochran–Armitage trend test where applicable. Ninety‐five percent confidence intervals for mortality risk were calculated using the Wilson score method; confidence intervals for Kaplan–Meier survival probabilities were generated directly by TriNetX's Analyze Outcomes module. Cochran–Armitage trend test for mortality risk and shifts in proportions of female patients were calculated in Python. Cell counts below 10 are suppressed by TriNetX and displayed as “≤ 10” to protect patient privacy.

### 2.6 Ethical Considerations

Only de‐identified, aggregate TriNetX output was used, and no patient‐level data were accessed. Such analyses are exempt from IRB oversight.

### 2.7 Artificial Intelligence Disclosure

An AI language model (Claude, Anthropic) assisted with organizing query outputs and drafting portions of the manuscript text based on results and interpretations provided by the author. All AI‐assisted output was reviewed and verified by the author, who takes full responsibility for its accuracy.

## 3 Results

### 3.1 Cohort Characteristics

The final analytic cohort comprised 3744 pediatric patients (age ≤ 17 years) undergoing TPE across the TriNetX US Collaborative Network (Table 1). Median age at index ranged from 0 years (IQR 1) in the youngest stratum to 15 years (IQR 2) in the oldest.

**TABLE 1:** Baseline demographic characteristics by age stratum.

Characteristic	≤ 1 year (*N* = 399)	2–5 years (*N* = 625)	6–12 years (*N* = 1172)	13–17 years (*N* = 1649)	All ages (*N* = 3744)
Age					
Median age at index, years (IQR)	0 (1)	3 (2)	10 (3)	15 (2)	11 (10)
Mean age at index ± SD, years	0.44 ± 0.50	3.48 ± 1.11	9.37 ± 1.99	15.1 ± 1.43	9.93 ± 5.50
Sex					
Female	176 (44%)^a^	281 (45%)^a^	586 (50%)^a^	910 (55%)^a^	1897 (51%)
Male	223 (56%)	344 (55%)	585 (50%)	739 (45%)	1846 (49%)
Ethnicity					
Not Hispanic or Latino	284 (72%)	406 (65%)	775 (66%)	1108 (67%)	2500 (67%)
Hispanic or Latino	80 (20%)	178 (29%)	314 (27%)	414 (25%)	962 (26%)
Race					
White	227 (57%)	352 (57%)	636 (54%)	889 (54%)	2048 (55%)
Black or African American	61 (15%)	101 (16%)	248 (21%)	379 (23%)	768 (21%)
Asian	13 (3%)	30 (5%)	64 (5%)	54 (3%)	156 (4%)
American Indian or Alaska Native	≤ 10 (3%)	≤ 10 (2%)	18 (2%)	21 (1%)	54 (1%)
Native Hawaiian or other Pacific Islander	≤ 10 (3%)	≤ 10 (2%)	12 (1%)	20 (1%)	41 (1%)

*Note:* Denominators reflect patients with an index event in the past 20 years for the outcomes analysis and may differ slightly from the raw query cohort size. Percentages are of the column *N* and may not sum to 100% due to rounding or unknown/missing values. Cell counts of ≤ 10 reflect TriNetX small‐cell suppression (true count is 1–10). All‐cohort and stratum‐level TriNetX queries were run independently and may reflect minor real‐time count variation; all‐cohort totals are reported as returned rather than as the sum of strata.

^a^ A Cochran–Armitage trend test confirmed a significant increase in the proportion of female patients with increasing age (*p* < 0.0001).

Sex distribution varied meaningfully across age strata. The two youngest strata showed a slight male predominance (≤ 1 year: 56% male vs. 44% female; 2–5 years: 55% male vs. 45% female), which attenuated to near parity in the 6–12‐year stratum (50% male, 50% female) and then reversed in the 13–17‐year stratum, where females predominated (55% female vs. 45% male). This male‐to‐female reversal across childhood and adolescence is a notable demographic feature of the cohort. A Cochran–Armitage trend test confirmed a significant increase in the proportion of female patients with increasing age (*p* < 0.0001).

All four age strata were predominantly White (54%–57% across strata) and non‐Hispanic/non‐Latino (65%–72% across strata), with Black or African American patients representing the next‐largest racial group (15%–23%, increasing somewhat with age) and Asian, American Indian/Alaska Native, and Native Hawaiian/Other Pacific Islander patients each representing small minorities (≤ 5% each stratum).

### 3.2 Indications by ASFA Category

Diagnosis codes recorded within 30 days of the index TPE procedure were mapped to ASFA Categories I–III indications (Table 2). Across the full cohort, the most frequently observed specific indication codes were autoimmune encephalitis (ICD‐10 G04.8; 393 patients, 10%), sepsis with multiorgan failure (R65.2; 344 patients, 9%), renal transplant rejection (T86.11; 213 patients, 6%), acute disseminated encephalomyelitis (ADEM) (G04.0; 201 patients, 5%), and Guillain–Barré syndrome (G61.0; 199 patients, 5%), followed by myasthenia gravis (G70; 136 patients, 4%). These six codes accounted for the large majority of specific‐indication coding across all four age strata.

**TABLE 2:** TPE indications by ASFA category and age stratum.

Indication	ICD‐10	≤ 1 year (*N* = 399)	2–5 years (*N* = 625)	6–12 years (*N* = 1172)	13–17 years (*N* = 1649)	All ages (*N* = 3744)
ASFA Category I						
TTP/TMA	M31.1	13 (3%)	18 (3%)	28 (2%)	64 (4%)	120 (3%)
Myasthenia gravis (acute)	G70	≤ 10 (3%)	16 (3%)	47 (4%)	76 (5%)	136 (4%)
Guillain–Barré syndrome	G61.0	13 (3%)	41 (7%)	58 (5%)	85 (5%)	199 (5%)
CIDP	G61.81	≤ 10 (3%)	≤ 10 (2%)	12 (1%)	12 (1%)	29 (1%)
Anti‐GBM disease	M31.0	≤ 10 (3%)	≤ 10 (2%)	17 (1%)	11 (1%)	29 (1%)
Recurrent FSGS (transplant)	N04.1	≤ 10 (3%)	≤ 10 (2%)	18 (2%)	22 (1%)	45 (1%)
Wilson disease (fulminant)	E83.01	0	0	≤ 10 (1%)	≤ 10 (1%)	≤ 10 (0%)
Atypical HUS	D59.39	≤ 10 (3%)	≤ 10 (2%)	≤ 10 (1%)	≤ 10 (1%)	33 (1%)
Hyperviscosity (myeloma)	C90.0	0	0	0	≤ 10 (1%)	≤ 10 (0%)
Renal transplant rejection	T86.11	0	31 (5%)	78 (7%)	124 (8%)	213 (6%)
ASFA Category II						
ADEM	G04.0	12 (3%)	63 (10%)	99 (8%)	32 (2%)	201 (5%)
MS (acute relapse)	G35	0	≤ 10 (2%)	33 (3%)	90 (5%)	127 (3%)
SLE (glomerular)	M32.14	0	0	16 (1%)	30 (2%)	45 (1%)
SLE (unspecified)	M32.10	0	0	12 (1%)	23 (1%)	33 (1%)
SLE (other organ)	M32.19	0	0	≤ 10 (1%)	11 (1%)	20 (1%)
NMOSD	G36.0	0	≤ 10 (2%)	43 (4%)	49 (3%)	92 (2%)
Cryoglobulinemia	D89.1	0	0	≤ 10 (1%)	0	≤ 10 (0%)
ASFA Category III						
Sepsis with multiorgan failure	R65.2	68 (17%)	71 (11%)	80 (7%)	126 (8%)	344 (9%)
HLH	D76.1	16 (4%)	16 (3%)	18 (2%)	24 (1%)	74 (2%)
HLH (infection‐associated)	D76.2	≤ 10 (3%)	≤ 10 (2%)	≤ 10 (1%)	≤ 10 (1%)	≤ 10 (0%)
Autoimmune encephalitis	G04.8	30 (8%)	63 (10%)	147 (13%)	155 (9%)	393 (10%)
Stiff‐person syndrome	G25.82	0	0	≤ 10 (1%)	≤ 10 (1%)	≤ 10 (0%)
Dermatomyositis/polymyositis	M33	0	≤ 10 (2%)	≤ 10 (1%)	≤ 10 (1%)	12 (0%)
ANCA vasculitis (GPA)	M31.3	≤ 10 (3%)	0	16 (1%)	29 (2%)	46 (1%)
ANCA vasculitis (MPA)	M31.7	0	≤ 10 (2%)	11 (1%)	18 (1%)	32 (1%)
Autoimmune hemolytic anemia	D59.1	≤ 10 (3%)	≤ 10 (2%)	15 (1%)	22 (1%)	52 (1%)
Liver transplant rejection	T86.41	17 (4%)	13 (2%)	≤ 10 (1%)	19 (1%)	57 (2%)
Nonspecific codes (not ASFA‐mapped)						
Other disorders of brain	G93	126 (32%)	201 (32%)	315 (27%)	346 (21%)	976 (26%)
Acute kidney failure/CKD	N17–N19	156 (39%)	203 (32%)	357 (30%)	520 (32%)	1201 (32%)
Complications of transplanted organs/tissue (general)	T86	41 (10%)	86 (14%)	177 (15%)	254 (15%)	522 (14%)

*Note:* Values are patients with the listed ICD‐10 code 30 days prior to the TPE procedure code, *N* (% of stratum). Patients may carry more than one code, so columns do not sum to the stratum *N*. Cell counts of ≤ 10 reflect TriNetX small‐cell suppression (true count is 1–10). ASFA categories reflect the Ninth Special Issue [2], the current edition at time of writing. All‐cohort and stratum‐level TriNetX queries were run independently and may reflect minor real‐time count variation; all‐cohort totals are reported as returned rather than as the sum of strata.

By contrast, several ASFA‐mapped codes were rare or entirely absent in one or more strata. Cryoglobulinemia, Wilson disease, hyperviscosity syndrome due to myeloma, and stiff‐person syndrome were each absent (zero patients) in at least two of the four age strata, and systemic lupus erythematosus–related codes (M32.10, M32.14, M32.19) were entirely absent in the ≤ 1‐year and 2–5‐year strata, consistent with the expected adolescent/adult‐predominant onset of SLE.

Nonspecific diagnosis codes were common across all strata and, notably, individually more prevalent than any single specific ASFA‐mapped indication. Acute kidney failure/chronic kidney disease (N17–N19) was present in 30%–39% of patients depending on stratum (1201 patients overall, 32%); other disorders of brain (G93) in 21%–32% of patients (976 patients overall, 26%); and general transplant‐organ complications (T86) in 10%–15% of patients (522 patients overall, 14%).

To assess whether the preprocedure‐only window artificially deflated specific‐indication capture, we performed a sensitivity analysis. We re‐ran the indication query including the day of the index procedure for the ≤ 17 cohort; the same pattern held, with nonspecific codes remaining more prevalent than any single ASFA‐mapped indication (data not shown).

### 3.3 All‐Cause Mortality

All‐cause mortality increased with duration of follow‐up and decreased monotonically with increasing age at every time window assessed (Table 3). At 30 days, mortality risk ranged from 16.8% in the ≤ 1‐year stratum down to 4.3% in the 13–17‐year stratum. This inverse relationship between age and mortality risk persisted at 90 days (25.1% vs. 5.7%) and at 1 year (30.1% vs. 7.7%), with the 6–12‐year and 13–17‐year strata tracking closely to one another at every timepoint (30‐day: 4.8% vs. 4.3%; 90‐day: 5.8% vs. 5.7%; 1‐year: 7.8% vs. 7.7%). A Cochran–Armitage trend test confirmed a significant monotonic decrease in mortality risk with increasing age at all three time windows (30‐day, 90‐day, 1‐year; all *p* < 0.0001).

**TABLE 3:** All‐cause mortality by age stratum and time window.

Age stratum	*N*	30‐day mortality			90‐day mortality			1‐year mortality		
		Deaths	Risk (95% CI)	KM survival (95% CI)	Deaths	Risk (95% CI)	KM survival (95% CI)	Deaths	Risk (95% CI)	KM survival (95% CI)
≤ 1 year	399	67	16.8%^a^ (13.4–20.8)	82.9% (78.9–86.3)	100	25.1%^a^ (21.1–29.5)	74.3% (69.7–78.4)	120	30.1%^a^ (25.8–34.7)	68.7% (63.8–73.1)
2–5 years	625	40	6.4%^a^ (4.7–8.6)	93.5% (91.3–95.2)	63	10.1%^a^ (8.0–12.7)	89.7% (87.0–91.9)	87	13.9%^a^ (11.4–16.9)	85.5% (82.4–88.1)
6–12 years	1172	56	4.8%^a^ (3.7–6.2)	95.2% (93.8–96.3)	68	5.8%^a^ (4.6–7.3)	94.1% (92.6–95.3)	91	7.8%^a^ (6.4–9.4)	92.0% (90.2–93.4)
13–17 years	1649	71	4.3%^a^ (3.4–5.4)	95.6% (94.5–96.5)	94	5.7%^a^ (4.7–6.9)	94.1% (92.9–95.2)	127	7.7%^a^ (6.5–9.1)	91.8% (90.3–93.1)
All ages	3744	234	6.3%^a^ (5.5–7.1)	93.7% (92.8–94.4)	324	8.7%^a^ (7.8–9.6)	91.2% (90.2–92.0)	421	11.2%^a^ (10.3–12.3)	88.2% (87.1–89.2)

*Note:* Risk = patients with outcome/patients in cohort, reported with 95% Wilson score confidence intervals in parentheses. KM survival = Kaplan–Meier survival probability at the end of the stated time window, reported with TriNetX‐derived 95% confidence intervals in parentheses. Outcome definition: Deceased (demographic flag) or ICD‐10‐CM R99 (Ill‐defined and unknown cause of mortality). Cohort denominators reflect patients meeting demographic‐completeness criteria for the outcomes analysis and may differ slightly from raw query cohort sizes reported elsewhere. All‐cohort and stratum‐level TriNetX queries were run independently and may reflect minor real‐time count variation; all‐cohort totals are reported as returned rather than as the sum of strata.

^a^ A Cochran–Armitage trend test confirmed a significant monotonic decrease in mortality risk with increasing age at all three time windows (30‐day, 90‐day, 1‐year; all *p* < 0.0001).

Within the ≤ 1‐year stratum, the increase in mortality risk was concentrated in the first 90 days following the index TPE procedure: Risk rose 8.3 percentage points between 30 and 90 days (16.8%–25.1%), compared with a further increase of only 5.0 percentage points between 90 days and 1 year (25.1%–30.1%). This early concentration of risk was proportionally much larger than in the older strata, where the 30–90 day increase ranged from only 1.0 to 3.7 percentage points. Kaplan–Meier survival probability in the ≤ 1‐year stratum fell from 82.9% at 30 days to 74.3% at 90 days to 68.7% at 1 year, underscoring that infants who survive the immediate post‐TPE period continue to accrue meaningful mortality risk over the following year, but at a substantially slower rate than in the first 3 months.

## 4 Discussion

This analysis encompasses 3744 patients across four age strata drawn from the TriNetX US Collaborative Network, and it surfaces three findings that together shape how this cohort should be interpreted. First, we observed a striking and consistent age gradient in all‐cause mortality, with infants (≤ 1 year) experiencing two‐ to fourfold higher mortality risk than every other pediatric age group at every time window assessed. This trend toward higher mortality risk with younger age was statistically significant (*p* < 0.0001). Moreover, infant mortality risk was concentrated disproportionately in the first 90 days following the index procedure. This pattern likely reflects an especially high‐acuity population rather than reflecting a TPE‐specific risk. Second, the sex distribution shifted from a slight male predominance in infancy and early childhood to a clear female predominance in adolescence, a statistically significant pattern (*p* < 0.0001) consistent with the known epidemiology of autoimmune disease onset. Several ASFA‐mapped indications identified in this cohort, including SLE and myasthenia gravis, disproportionately affect females with onset around or after puberty, consistent with population‐based pediatric SLE epidemiology showing a roughly fivefold higher prevalence in girls and a marked rise in incidence after early adolescence [18]. Third, nonspecific diagnosis codes were each individually more prevalent than any single ASFA‐mapped specific indication. This could potentially reflect a practice of starting empiric TPE before the diagnostic work‐up is complete, and we note that we only assessed a 30‐day preprocedure window for indication codes. Nonetheless, this pattern has direct implications for claims‐based indication‐assignment methodology in future TriNetX‐based apheresis studies.

Despite this nonspecific coding burden, the specific indications that were captured align with clinical expectation: Kidney‐related (renal transplant rejection) and neurologic (autoimmune encephalitis, ADEM, Guillain–Barré syndrome, myasthenia gravis) indications were the most common specific reasons for pediatric TPE across nearly every age stratum. Notably, several of the most prevalent specific codes are conditions with a recognized pediatric predominance or pediatric‐specific clinical significance: ADEM is classically a childhood‐onset demyelinating disorder [19], atypical hemolytic uremic syndrome and recurrent FSGS after transplantation are both well‐described pediatric nephrology entities [20], and autoimmune encephalitis (including anti‐NMDA‐receptor encephalitis, which is identified more frequently in children than any single viral cause of encephalitis) is a leading cause of pediatric encephalitis [21]. The prominence of these pediatric‐specific entities in our indication data, rather than indications more typical of adult apheresis populations, supports the face validity of the cohort‐identification and indication‐mapping approach used in this analysis.

These findings can be placed directly alongside those of Weiss et al., the only prior multi‐institutional pediatric TPE study and thus our closest methodological comparator [16]. Weiss et al. examined the same underlying practice gap from two angles. First, starting from an administrative, diagnosis‐defined cohort, they asked how often those children received TPE, and reported low, hospital‐variable utilization: 13.4% of Category I and 9.3% of Category II hospitalizations. Second, and more directly comparable to our own approach, they also characterized all hospitalizations with a TPE procedure code regardless of diagnosis, and found that only 9.0% and 2.2% of these carried a primary discharge diagnosis of a Category I or II condition, respectively, with complications of a transplanted heart or kidney and hemolytic‐uremic syndrome the leading alternative discharge diagnoses. This procedure‐first result anticipates our own central finding: Across two independent, decade‐apart, multi‐institutional US datasets, the large majority of real‐world pediatric TPE use occurs outside a strict ASFA Category I/II framework. Our data extend this observation by explicitly capturing Category III indications and by showing that nonspecific diagnosis codes (AKI/CKD, other disorders of brain, general transplant complications) individually outnumber any single ASFA‐mapped code. The roughly 15‐year gap between the two datasets is also relevant: several ASFA category reassignments occurred in the interim (e.g., ANCA‐associated vasculitis was downgraded from Category I to Category III in the 2023 ASFA guideline update), and the transition from ICD‐9‐CM to ICD‐10‐CM further limits direct code‐level comparability between the two studies. Finally, Weiss et al. did not report age‐stratified or long‐term mortality outcomes beyond a small, underpowered sensitivity analysis restricted to TTP (seven deaths total); the age‐stratified 30‐day, 90‐day, and 1‐year mortality estimates presented here extend, rather than duplicate, that earlier multi‐institutional effort.

These findings can also be placed alongside the broader pediatric TPE literature (Table 4), which, consistent with the gap motivating this study, consists almost entirely of single‐ or few‐center cohorts an order of magnitude smaller than ours [3, 4, 5, 6, 7, 8, 9, 10, 11, 12, 13, 14]. Across most general‐PICU cohorts, sepsis/MODS was the single most common indication and the large majority of indications were ASFA Category III; sepsis/MODS appears in our cohort at a lower rate (9%) and is not the leading specific indication, consistent with a broader, less acuity‐selected sampling frame. Cohorts restricted to pediatric neurology referral populations diverge from the PICU series, with inflammatory polyneuropathy, ADEM, and NMOSD predominating. The specific indications most prevalent in our cohort—autoimmune encephalitis, renal transplant rejection, ADEM, and GBS—recur throughout the PICU literature as well, lending reasonable external validity to our indication‐mapping approach despite very different sampling strategies. Together, these contrasts illustrate how strongly referral pattern and case selection, rather than TPE itself, shape the reported indication mix and mortality range (4.3%–50% across the series synthesized by Ozel et al.). Reported mortality generally tracked illness‐severity scores rather than any TPE‐specific factor, consistent with our own conclusion that mortality reflects underlying acuity. Because our cohort captures all pediatric TPE recipients across a large network regardless of ICU status, it is substantially broader than these PICU‐restricted series—likely explaining why our all‐ages 1‐year mortality (11.2%) sits below their range, while our youngest stratum (30.1%) falls within or above it. Tandem‐extracorporeal‐therapy series (TPE with CRRT/ECMO or CPB) report consistently elevated mortality (21%–48%), which we could not directly compare our cohort to since TriNetX does not capture concurrent CRRT/ECMO use, but which reinforces that combined extracorporeal support marks a particularly high‐acuity subset.

**TABLE 4:** Synthesis of pediatric TPE literature.

Study	Country	Setting	*N*	Leading indication(s)	ASFA category mix	Mortality
Bustos et al. [7]	Chile	General PICU	36	Autoimmune neurologic disease (38.9%); renal disease second	Cat I–II 44.4%; Cat III 50%	8% (PICU)
Atay & Demirkol [5]	Turkey	Mixed PICU	39	MAS (28.2%); renal graft rejection and liver failure (15.4% each); GBS (15%)	Cat I ~38.5%; Cat III ~56.4%	Not reported
Haque et al. [4]	Pakistan	PICU (92% of cases)	28	Neurologic illness (46%, incl. GBS *n* = 10); TTP/HUS (32%)	Cat I 81%; Cat II 15%; Cat III 4%	17.9% (5/28)
Özkale et al. [3]	Turkey	Neurology‐referral PICU	22	Inflammatory polyneuropathy/GBS; ADEM	Not reported (by‐diagnosis only)	0% TPE‐attributable; 1 unrelated death (e.g., sepsis)
Sık et al. [6]	Turkey	General PICU	135	Sepsis/MODS (44.4%); hematologic (19.2%); neurologic (9.6%)	Cat III 71.1%, Cat I 15.6%, Cat II 7.5%	21.5% (100%–78.5% survival)
Shamarao et al. [8]	India	General PICU	33	Acute liver failure (48.5%); sepsis/MODS second	Cat I 57%; Cat II 21%; Cat III 15%	40.7% (100%−59.3% survival)
Durak et al. [9]	Turkey	General PICU	102	COVID‐19‐related conditions; sepsis (24.5%)	Not reported (by‐diagnosis only)	13.7%
Bingol et al. [10]	Turkey	General PICU	83	Sepsis/MODS (65.1%); neurologic (9.6%); renal (8.4%)	Cat I 8.4%; Cat II 9.6%; Cat III 81.9%	26.5% (22/83)
Ozel et al. [11]—synthesis of 16 series	Multiple (mostly Turkey)	General PICU (aggregated)	24–328 per series	Sepsis/MODS most common across series	Predominantly Cat III	4.3%–50% across series
Sirignano et al. [12]—tandem	United States	ICU, tandem CVVH/CPB/ECMO	53	Solid organ transplant (63%); sepsis‐induced MOF (26%)	~83% outside Cat I/II	21%
Tufan Pekkucuksen et al. [13]—tandem	United States	ICU, tandem TPE + CKRT	63	Acute liver failure (31.7%); TAMOF (30.2%)	90% Cat III, 3% Cat II, 6% Cat I	40%
Aplenc et al. [14]—tandem	United States	ICU, tandem TPE + other extracorporeal circuits	50	Sepsis/MOF (22%); MOF (18%)	Majority Cat III	48% at 30 days (52% survival)

As with any large administrative database analysis, this study's design shapes how its findings should be read. It is a retrospective, code‐based landscape analysis without adjustment for confounders, comorbidity burden, or illness severity—the mortality figures reported here describe the cohort rather than establish TPE‐attributable risk. Indication assignment relied on ICD‐10‐CM codes within a 30‐day preprocedure window rather than chart‐confirmed diagnoses, and several codes (e.g., atypical HUS) span more than one ASFA category, so the ASFA distributions reported here are approximate. A sensitivity analysis using diagnosis codes from the day of the index procedure reproduced the same pattern of nonspecific‐code predominance, confirming this is not an artifact of the 30‐day window. However, later diagnostic refinement captured only in discharge coding, which TriNetX's aggregate structure does not expose, remains a possibility. TriNetX's small‐cell suppression limits precision for rarer indications, and because the mortality outcome does not capture cause of death, we cannot separate TPE‐related complications from disease progression or unrelated causes. Coding practices and thresholds for pursuing TPE likely vary across the participating HCOs in ways a network‐level analysis cannot resolve. Finally, because our outcome was restricted to all‐cause mortality, this analysis does not address procedural complication rates; prior single‐center cohorts report major complication rates of roughly 2%–4% [3, 4], with considerably higher perprocedure rates in cohorts using broader complication definitions (7%–55%) [15]. Future TriNetX‐based work incorporating adverse‐event coding can extend the estimates presented here.

Several directions for future work follow from these findings. First, the infant mortality pattern warrants dedicated study. Of the 120 deaths within 1 year in the ≤ 1‐year stratum, 67 (56%) occurred within 30 days of the index procedure and 100 (83%) within 90 days, indicating that risk is concentrated in the immediate periprocedural period. Future analyses could separate neonates from older infants and examine mortality by underlying indication (sepsis with multiorgan failure, for example, was coded in 17% of infants versus 7%–11% of older children). They could also incorporate proxies for illness severity to test whether early mortality reflects acuity, as suggested here, or varies by indication or timing of TPE initiation. Matching infants who received TPE to infants with the same diagnoses who did not would be required to distinguish procedure‐attributable from disease‐attributable risk. Second, the shift from male to female predominance across age strata was not cross‐tabulated by indication in this analysis. Sex‐stratified analyses within individual indications, using the number of patients carrying each diagnosis as the denominator, could determine whether the adolescent female predominance simply tracks the sex distribution of underlying autoimmune disease or instead reflects differences in referral or TPE utilization, and whether outcomes differ by sex. Third, given that TPE may be initiated before a diagnosis is established, a natural next step is to compare outcomes between patients with an ASFA Categories I and II‐mapped diagnosis (indication‐led) and those with only nonspecific or Category III codes (empiric). Endpoints beyond mortality, such as the number of TPE procedures, subsequent healthcare utilization, and coded complications, would better capture potential benefit, since mortality alone cannot establish benefit without an untreated comparator. Confounding by indication, overlapping code assignments, and the limited ability of diagnosis codes to distinguish empiric from indication‐led use would require explicit handling. Finally, these age‐stratified estimates can inform sample‐size calculations and the design of prospective multicenter pediatric apheresis registries.

## 5 Conclusion

In this multi‐institutional analysis of 3744 pediatric patients undergoing TPE across the TriNetX US Collaborative Network, we identified a consistent, steep inverse relationship between age and all‐cause mortality, with infants younger than 1 year experiencing two‐ to fourfold higher mortality risk than older children and adolescents at 30 days, 90 days, and 1 year. This mortality risk was concentrated disproportionately within the first 90 days after the index procedure. A shift from male predominance in infants and young children to female predominance in adolescents was noted, consistent with the known epidemiology of autoimmune disease onset. Finally, we found a predominance of nonspecific diagnosis codes over any single specific ASFA‐mapped indication, potentially reflecting the timing of diagnostic workup relative to TPE initiation rather than the true underlying distribution of indications. Despite this coding limitation, the specific indications identified—dominated by neurologic and renal etiologies, several of which carry recognized pediatric‐specific significance—aligned well with established clinical practice. These findings provide age‐stratified mortality estimates that may be useful for clinical counseling, quality benchmarking, and hypothesis generation, while underscoring that they cannot establish TPE‐attributable risk.

## Funding

The author has nothing to report.

## Ethics Statement

The author has nothing to report.

## Conflicts of Interest

The author declares no conflicts of interest.

## Data Availability

The data that support the findings of this study are available from TriNetX. Restrictions apply to the availability of these data, which were used under license for this study. Data are available from https://live.trinetx.com with the permission of TriNetX.
